# Use of Insecticide-Treated Mosquito Net among Pregnant Women and Guardians of Children under Five in the Democratic Republic of the Congo

**DOI:** 10.1155/2017/5923696

**Published:** 2017-11-06

**Authors:** Joseph N. Inungu, Nestor Ankiba, Mark Minelli, Vincent Mumford, Dido Bolekela, Bienvenu Mukoso, Willy Onema, Etienne Kouton, Dolapo Raji

**Affiliations:** ^1^Central Michigan University, Mount Pleasant, MI, USA; ^2^Association de Santé Familiale, Kinshasa, Democratic Republic of the Congo; ^3^Centre Africain de Recherche et Développement, Kinshasa, Democratic Republic of the Congo; ^4^Centre de Recherche et d'Appui-Conseil pour le Développement, Benin

## Abstract

**Background:**

Insecticide-treated mosquito nets (ITNs) are one of the most effective tools for preventing malaria in sub-Saharan Africa.

**Objective:**

This study examined knowledge, attitude, and practice on the use of ITNs in the prevention of malaria among pregnant women and guardians of children under five in the Democratic Republic of the Congo.

**Methods:**

A total of 5,138 pregnant women and guardians of children under five were interviewed.

**Results:**

The majority of participants (>80%) knew the signs and symptoms of malaria; 81.6% reported having an ITN in the household, but 78.4% reported using it the night before the interview. Only 71.4% of pregnant women used ITN the night compared to 68.2% of children under five. In the Logistic Regression model, women who believed that it is normal to use ITNs were 1.9 times more likely to use it than those who did not (OR: 1.930); women who were confident in their abilities to use ITNs were 1.9 times more likely than those who were not confident (OR: 1.915); and women who had a good attitude towards ITNs were also more likely to use ITNs compared to those who did not (OR: 1.529).

**Conclusion:**

New and innovative evidence-based behavior change interventions are needed to increase the utilization of ITNs among vulnerable groups.

## 1. Background

Malaria is a major cause of mortality and morbidity in the world, accounting for 148–304 million cases and an estimated 235,000–639,000 deaths globally. Most deaths occurred in the World Health Organization (WHO) African Region (92%), followed by the WHO South-East Asia Region (6%) and the WHO Eastern Mediterranean Region (2%). Between 165,000 and 450,000 deaths are estimated to have occurred in children aged under 5 years, which is equivalent to 70% of the global total [1].

Malaria is caused by an intraerythrocytic protozoa of the genus* Plasmodium* transmitted by the bite of an infective female* Anopheles* mosquito. Of the four Plasmodium species that infect humans,* P. falciparum* is common in sub-Saharan Africa and causes severe and potentially fatal malaria [1]. The other 3 species include* P. vivax, P. ovale*, and* P. malariae. P. vivax* is common in Asia and Latin America [2]. Malaria can also be transmitted through transfusion of contaminated blood or during child birth [3].

The signs and symptoms of malaria illness vary widely. Symptoms of uncomplicated malaria episode include fever, chills, sweats, headache, nausea, vomiting, body ache, and malaise, whereas symptoms of severe clinical episode are organ dependent. Abnormal behavior, seizures, coma, or impairment of consciousness is observed in cerebral malaria. Anemia, jaundice, and hemoglobinuria result from massive destruction of red blood cells. Other severe symptoms include acute respiratory distress, low blood pressure, acute kidney failure, metabolic acidosis, and hypoglycemia [4]. Conventional diagnosis is based on examination of thin and thick blood films stained with Giemsa's or Field's stain. The antigen-based dipstick tests known as “Rapid Diagnostic Tests” (RDTs) offer a useful alternative to microscopy in situations where reliable microscopic diagnosis is not available [4]. PCR-based diagnostic tests for human malarias are more applicable to large-scale surveys than to clinical diagnosis [5].

In addition to the human cost, the economic burden of malaria is vast. It is estimated that malaria costs African countries more than US $12 billion every year in direct losses [6]. This figure includes the costs of healthcare, absenteeism, days lost in education, decreased productivity due to brain damage from cerebral malaria, and loss of investment and tourism. Certain countries that managed to completely eliminate malaria in recent times have had more rapid economic growth than their neighbors [7].

The Government of the Democratic Republic of Congo (DRC), through the National Malaria Control Program (NMCP), has, with the support of its partners (including the World Bank, USAID, UNICEF, Association for Family Health (ASF) and the Global Fund), relied on the use of insecticide-treated mosquito nets (ITNs), together with treatment of malaria with artemisinin-based combination therapy (ACT) to control malaria in the country. However, access to ACT is difficult in the DR Congo. Due to high cost, ACT is not readily available in the private sector [8]. Counterfeiting of ACT is a growing global challenge [9]. In addition, report of the emergence of artemisinin-resistant* P. falciparum* in Africa is very disturbing [10]. Efforts by the government of the Congo to scale up free distribution of ITNs during the last 10 years contributed to increase household ITNs ownership rates to 72% although ITNs utilization remains low (52%) [11]. Modern mosquito nets lack physical durability, and household nets can accrue an average of 12–20 holes during 1-2 years of use [12]. Net replacement schemes struggle to meet demand at this level of deterioration and attrition [13]. The objective of this study was to examine knowledge, attitude, and practice about the use of insecticide-treated mosquito nets (ITNs) in the prevention of malaria among pregnant women and guardians of children under five in the Democratic Republic of the Congo.

### 1.1. Theoretical Framework

**Figure 1:**
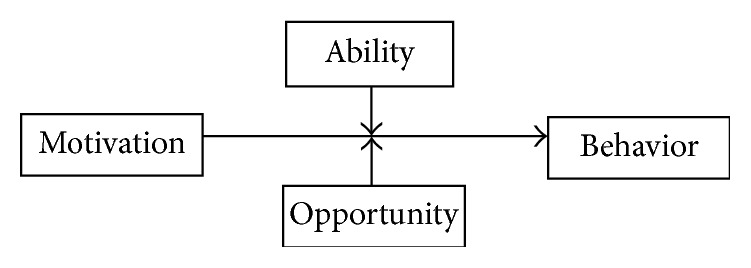
Opportunity, motivation, and ability (OMA) framework.

Although there are multiple frameworks, approaches, and models for understanding behavior and designing related interventions [14–16], opportunity, motivation, and ability (OMA) framework was used to guide the selection of variables for this study. OMA framework draws upon consumer behavior, social marketing, and health behavior theories [17]. It assembles basic concepts of psychology: motivation, the impetus towards a behavior; ability, skills and capabilities requisite to the performance of a behavior; and opportunity, contextual and situational constraints relevant to the performance of the behavior.  Figure 1 depicts the relationship between opportunity, ability, and motivation. MacInnis and Jaworski theorized that both ability and opportunity as moderators of the impulse towards a particular behavior triggered by motivation [18]. OMA framework was previously used in research dealing with nutrition [19], water, sanitation, hygiene sector [20], and global health [21].

## 2. Methods

### 2.1. Sampling Approach

This is a population-based household cross-sectional survey undertaken in 8 of the 11 provinces in the Democratic Republic of the Congo in 2015. The other 3 provinces were not included because they were involved in a pilot study on the use of insecticide-treated mosquito nets in 2014. Multistage cluster sampling design was used to select a representative sample of pregnant women and/or guardians of children under 5 years living in these households.

First, 177 health areas (HA) were selected out of 5624 using a probability proportional to size (PPS) sampling method, so that more populated HAs had a higher probability of being selected. In each HA, households with at least one child aged 6–59 months or a pregnant woman were listed by community health workers (CHW). From the list of households, 35 eligible households were randomly selected, yielding a total of 5748 households. One guardian or pregnant woman was interviewed in each household.

Households were revisited if no one was available for interview on the first attempt; if no one was available after three attempts, the interviewer continued to the next randomly selected household on the list until the desired number of households was obtained. Households where eligible participants refused to be interviewed were excluded.

### 2.2. Data Collection

Eight teams of 15 enumerators and 3 community liaisons were trained in interviewing techniques and administration of informed consent during simulated interviews sessions. A pretested questionnaire was used to collect information. The questionnaire was developed in French with oral translation into locale languages (Lingala, Kikongo, Swahili, and Tshiluba) and field tested prior to the survey. At each household, fieldworkers introduced themselves and explained the purpose of the study. An informed consent was sought from the respondent prior to administering the questionnaire. Participation in the study was entirely voluntary.

The questionnaire was designed to collect information on household characteristics, education status, family size, household amenities and assets, bednet ownership, source of bednets, bednet utilization, history of fever (past 2 weeks), factor of opportunity (perception of the bednet, cost, availability, social norm), factor of ability (knowledge about malaria, knowledge about mode of prevention, and self-efficacy), and factor of motivation (perception about severity, usefulness, and attitude towards the brand). To ensure consistency and integrity of data collected, 20% of the forms were rechecked by team supervisors in the field at the end of each day. Incomplete entries were sent back to be filled the next day. Questionnaires were first checked for completeness, and the information was manually coded.

### 2.3. Data Analysis

Data were entered using the Census Surveys Professional (CSPro) software and were exported to and analyzed using SPSS 22.0. Descriptive statistics were carried out for sociodemographic characteristics, ITN household ownership and utilization of bednets, and knowledge of malaria. Logistic regression analysis was used to assess the independent association between independent variables and the outcome of interest.

### 2.4. Dependent Variable

The use of bednets the night before the interview was the dependent variable for this study. All insecticide-treated mosquito nets, whether long-lasting insecticide treated nets or retreated nets, are referred to as ITNs.

### 2.5. Independent Variables

The independent variables included place of residence (urban or rural), age, education, knowledge of the cause of malaria, social norm, self-efficacy, perception about severity of malaria, and attitude towards ITN. We used the Amenities and Possession Index, a poverty/wealth indicator based on household access to 3 basic amenities (drinking water, toilet, and electricity) and 4 consumer durable possessions (radio, television, refrigerator, and car). A person was assigned to one of three categories (high, medium, and low) according to whether the household in which the person resides has access to different combinations of the following amenities and consumer goods: toilet facilities, drinking and nondrinking water, electricity, radio, television, refrigerator, and car. The method to compute the Amenities and Possession Index was described elsewhere [22].

Participants were asked to list all the signs or symptoms of malaria and name all possible methods used to prevent malaria. Based on the correct answers relative to the signs and symptoms of malaria, three groups were created: 0: “listed no correct sign or symptom”; 1-2: “listed 1 to 2 correct signs or symptoms”; and 3: “listed 3 or more signs or symptoms of malaria.” To assess participants' knowledge about the methods to prevent malaria, we created 3 groups: 0: “named no correct prevention method”; 1-2: “named 1 to 2 correct prevention methods”; and 3: “named 3 or more prevention methods.”

### 2.6. Ethical Consideration

Participants were informed about the purpose of the study. An informed consent was sought out before administering the questionnaire. Participation in this project was voluntary. Ethical clearance was obtained from the Ethics Committee of the School of Public Health in Kinshasa, University of Kinshasa.

## 3. Results

### 3.1. Sociodemographic Characteristics

Table 1 summarizes the sociodemographic characteristics of the participants. Of the 5,138 individuals who participated in the survey, 4,966 (96.6%) were women. Among them, 2,008 were pregnant at the time of the study. The majority of the participants (68.8%) were married and 35.5% of them were housewives. About 74.3% of participants had at least a primary level of education; 54.3% were 15 to 29 years of age. Catholic, protestant, and charismatic churches were the most commonly reported religious affiliations. The majority of participants lived in urban areas (61.6%), and had a low socioeconomic status (42.5%).

### 3.2. Malaria Knowledge among Participants

Participants in the study had a high level of knowledge about malaria. More than 80% of the participants knew a relative who suffered from malaria in the past 12 months, could name at least one sign or symptom of malaria, or list one method to prevent malaria (Table 2). Although the majority of participants knew that mosquito bites transmit malaria (89%), misconceptions about malaria transmission persist. Some respondents thought that drinking unclean water (19.1%) or being exposed to the sun (3.1%) could cause malaria. Health centers (47.2%), radio stations (24.7%), and community health workers (10.5%) were the most commonly reported sources of information about malaria.

### 3.3. Bednet Ownership and Utilization

Table 3 summarizes ITN ownership and utilization. ITN ownership rates were high. Eighty-one percent (81.6%) of the participants reported having an ITN in their household. About 75.7% reported having 2 or more bednets. ITN ownership varies among provinces. The Oriental Province (91.9%), Bas Congo (89.2%), and Kasai East (86.2%) reached universal coverage. The lowest coverage rate was reported in Kasai Occidental Province (67.9%) (Table 4).

Regarding the utilization of ITN, 78.4% participants reported having used an ITN the night before the interview. More than 80% of the participants in the Oriental Province, Bas Congo, and Kasai Oriental reported using ITN the night before the interview compared to 69.2 and 72% in the Kasai Occidental and Bandundu province, respectively (Table 4).

Among groups most vulnerable to malaria, 71.4% of pregnant women reported having slept under an ITN the night before the survey. The Oriental Province (81.6%) and Maniema (81.4%) reported the highest utilization rate compared to Kasai Occidental (64.5%), Bas Congo (66.4%), and even Kinshasa (67.7%). Guardians of children under five reported that 68.2% of children slept under ITN the night before the interview. The highest utilization rate was reported in Kasai Oriental (74.6%), Bas Congo (74.0%), and the Oriental Province (73.5%) compared to Maniema (59.2%) and Kasai Occidental (62.7%).

When asked if they would purchase a bednet if given money, 65% of participants answered affirmatively. They were further asked which bednet would they use between the one they purchased and the other received free of charge. The majority of participants (72%) said that the method of acquisition of ITN has no bearing on the decision to use it or not. Examining where people get information about bednets, we noted that the health centers (40%) and radio stations (20%) were the most commonly reported sources of information. Participants were asked to explain the reasons why they did not use an ITN the night before the interview. Lack of money and the unbearable heat were the two main reasons for not using bednets.

### 3.4. Determinants of ITN Utilization

Only independent variables found to be associated with the independent variable in the bivariate analysis were included in the Logistic Regression Model (Table 5). Of all the factors included in the model, age, marital status, and perception of severity were not significantly associated with the use of ITNs. Perception about ITN, self-efficacy, social norm, and attitude towards ITNs had a relatively strong association with ITN utilization. Women who believe that it is normal to use ITNs were 1.9 times more likely to use ITN than those who did not (OR: 1.930; 95% CI: 1.645–2.265). Women who were confident in their abilities to use ITNs were 1.9 times more likely than those who do not to use ITNs (OR: 1.915, 95% CI: 1.489–2.464). Women who had a good attitude towards ITNs were more likely to use ITNs compared to those who do not (OR: 1.529, 95% CI: 1.258–1.858).

Level of education, place of residence, and knowledge of the cause of malaria were associated with the use of ITNs, but the association was borderline. Women with high school education or higher were 1.3 times more likely than those with up to primary school level of education to use ITNs (OR = 1.3, 95% CI: 1.085–1.611). Women who knew that mosquito bites transmit the agent that causes malaria were significantly more likely those who did not know to use ITNs (OR: 1.378; 95% CI: 1.09–1.742). Women living in urban areas were more likely to use ITNS than those in rural areas (OR = 1.236; 95% CI: 1.049–1.458).

## 4. Discussion

This study was undertaken to examine knowledge, attitude, and practice on the use of insecticide-treated mosquito nets in the prevention of malaria among pregnant women and guardian of children under five in the Democratic Republic of the Congo. Considering that two-thirds of the population in the Democratic Republic of the Congo live in malaria endemic areas and ITN use is widely recognized as an effective intervention to prevent malaria, the National Malaria Control Program (PNLP), in partnership with international aid agencies, has been organizing mass distribution of ITNs across the country to increase ownership and promote utilization of ITNs. Ownership of at least one bednet per household is the targeted outcome of the campaign.

Our study showed rapid attainment of high ITN coverage through free bed net distribution in impoverished communities. A coverage rate of 81.6% was achieved in the 8 provinces of the DR Congo following free bed net distribution. High ITN coverage rates following free bed nets distribution were previously reported in the Congo [23] and elsewhere in sub-Saharan Africa including Sierra Leone (87.6%) [24], Ethiopia (91.0%) [24], and Togo (96.7%) [25]. However, the size of the DR Congo, being as large as the size of the United State West of Mississippi River, presents a daunting challenge for the NMCP to organize mass distribution of ITNs across the country. As a result, ITN coverage rates vary widely across the provinces. ITN ownership rate of 91.9% was reported in the Oriental Province compared 67.9% in the Kasai Occidental Province. More investments are needed to assist the National Malaria Control Program to scale up ITN distribution in selected provinces where the coverage remains low (e.g., Kasai Occidental Province).

This study also showed that high ITN coverage did not translate into concomitant high ITN utilization. Although the percentage of households that own at least one ITN was quite high (81.6%), the utilization of ITN on the other hand remained low among children under five (68.2%) and moderate among pregnant women (71.4%). While low utilization of bed nets among children under five were reported in previous studies [5, 16], the reasons behind that observation have not been elucidated. We posit that the difference in the sleeping arrangements of children under five could, in part, explain this result [26]. Baume and Marin reported that parents consider children under 2 years of age to be more vulnerable to malaria and tend to place them in priority under bed nets [27]. They are inclined to remove their 3-year-old child from the parents' bedroom to join their older siblings who often times spend the night in the living room or in the hall ways. Unlike the 2-year-old children, the older children are not protected and therefore more exposed to malaria. More studies are needed to conform this assertion.

Finally, this study examined the factors associated with ITN utilization and found that social norm, self-efficacy, women's perception about ITN, and factors of motivation (such as attitude towards bednet) were significantly associated with ITN utilization. These findings underscore the need for designing new, effective, and evidence-based behavior change interventions to enhance ITN utilization in the Congo. To improve the utilization rate of ITNs in the Congo, policy makers should consider using a multifaceted intervention addressing people's self-efficacy in placing bed nets in the bedrooms, the prevailing social norm in the community, and their attitude and acceptability of bed nets. Increasing people's ownership of bed net alone does not always translate into action. Furthermore, existing misconceptions about the cause of malaria may prevent community members from taking the right course of action to prevent malaria. For example, the belief that drinking uncleaned water or being exposed to the sun causes malaria can prevent people from using ITNs to protect themselves.

Considering the proven effectiveness of ITNs in reducing malaria morbidity and mortality, it is imperative for the NMCP to remain focused in promoting both ITN ownership and utilization since access to bed nets does not guarantee their utilization. Public debate has largely focused upon the comparative merits of free and market-based strategies for deploying ITNs [28–30]. While mass distribution has been shown to be the best approach to achieve rapid scale, it requires huge capital to organize and carry out the campaign. In addition, loss of physical integrity of ITNs and inaccessibility due to geographic barriers constitute major hindrances to maintaining universal coverage [31]. Considering that funding from donors will likely decrease over time, the commercial sector's ability to sell ITNs in retail markets appears to be a viable option. But experiences in many different countries show that only around 20% of the population is willing and able to pay for the nets. This coverage is not high enough for protecting vulnerable groups nor for achieving the “mass effect.” In a study conducted in Tanzania, Magesa et al. [32] achieved rapid attainment of high net coverage for the vulnerable population through the combined contributions of the product provision campaign and voucher subsidy while broad coverage for the rest of the community resulted largely from nets purchased on the open market at full price. The lesson learned from this study is to apply diverse approaches to deliver ITN simultaneously in an imperfect but constructive and complementary manner.

## 5. Limitations

The first limitation of this study is the lack of baseline data to assess the extent to which free bed net distribution improved ITN coverage in the 8 provinces surveyed. The use of self-reported information on ownership and utilization of ITNs without verification is prone to recall and information biases. Respondents could have reported ITN ownership and utilization due to social desirability. The strength of this study is its large sample size and the collection of data in several provinces of the Democratic Republic of the Congo.

## 6. Conclusion

Although mass distribution of ITNs contributed to high level of knowledge about malaria and attainment of rapid high ITN coverage rates in the 8 provinces studied, the utilization of ITNs among pregnant women and children under five remained low. The NMCP should take into account the factors associated with the use of ITNs in designing evidence-based behavior change interventions to improve the utilization of ITNs in the Democratic Republic of the Congo.

## Figures and Tables

**Table 1:** Demographic characteristics of the participants.

Characteristics	Description	Total	Percentage (%)
		*N* = 5138	
Education level	No education	1040	20.2
	Primary school level	1215	23.6
	Secondary school+	2552	49.7
	College	331	6.4

Age	15–19	472	9.3
	20–24	1055	20.8
	25–29	1227	24.2
	30–34	984	19.4
	35–39	691	13.6
	40–44	325	6.4
	45+	313	6.2

Marital status	Single	417	8.1
	Married	3502	68.8
	Married poly	378	7.4
	Divorced/widowed	148	2.9
	Widows	138	2.7
	Free union	509	10.0

Residence-urban	No	1971	38.4
	Yes	3167	61.6

Occupation	Housewife	1804	35.5
	No job	522	10.3
	Employees	415	8.2
	Farmer	1005	19.8
	Small business	669	13.2
	Others	660	13.0

Religion	Islam	159	3.1
	Catholic	1556	30.6
	Protestant	1064	20.9
	New movement	1795	35.3
	Kimbanguists	227	4.5
	Black Church	55	1.1
	Animists	8	0.2
	Others	221	4.3

Socioeconomic status^*∗*^	Low	2184	42.5
	Average	1479	28.8
	High	1475	28.7

^*∗*^Based on the Amenities and Possession Index.

**Table 2:** Malaria knowledge among participants.

Characteristics	Description	Frequency	Percentage
History of malaria in your family in the past 12 months	No	771	18.8
	Yes	3321	81.2

Number sign/symptoms of malaria known	0	87	1.9
	1-2	1101	23.5
	3 and more	3500	74.7

Number of methods to prevent malaria known	0	168	3.3
	1-2	470	9.1
	3 and more	4079	79.4

Knowledge of the cause of malaria	No	508	10.8
	Yes	4205	89.2

Best ways to protect against malaria	Keep house clean	1386	27.7
	Insecticide treated bednets	982	19.6
	Drink clean water/avoid drinking dirty water	980	19.1
	Spray insecticide	507	10.1
	Take medicine	390	7.8
	Avoid exposure to the sun	153	3.1
	Others	316	6.3

Sources of information	Health centers	1277	47.2
	Radio	669	24.7
	Health workers	284	10.5
	Television	221	8.2
	Posters	50	1.8
	Billboards	48	1.8
	Others	86	3.2

**Table 3:** Bednet ownership and utilization.

Characteristics	Description	Frequency	Percentage
Do you have a bednet in your household?	No	896	18.4
	Yes	3985	81.6

How many bednet do you have in the household?	1	953	24.3
	2	1231	31.4
	3	1018	25.9
	4+	721	18.4

Did you use the bednet last night?	No	1023	21.6
	Yes	3710	78.4

How often do you use it a week?	Everyday	3722	73.7
	2	181	3.6
	3	282	5.6
	Never	862	17.1

Have you ever bought a bednet	No	4468	88.3
	Yes	590	11.7

How much it cost you the last time you bought one?	$2.0 (sd: 720)		

Would you prefer the bednet you bought over the one you received for free	No	3638	72.1
	Yes	1406	27.9

If you had money, will you buy a bednet?	No	1793	35.4
	Yes	3278	64.6

Being counseled about bednet	No	1117	22.4
	Yes	3871	77.6

Where did you get information about bednets	Health centers	1520	40.4
	Radio	779	20.7
	Church	458	12.2
	CHW	301	8.0
	TV	225	6.3
	Neighbors	193	5.1
	Family	54	1.4
	Sellers	79	2.1
	Others	153	4.1

**Table 4:** ITN ownership and utilization among groups vulnerable in the different provinces.

	ITN ownership		ITN utilization					
			All participants		Pregnant women		Under five	
	No	yes	No	Yes	No	Yes	No	Yes
*Total*	*896 (18.4)*	*3985 (81.6)*	*971 (21.4)*	*3574 (78.6)*	*574 (28.6)*	*1434 (71.4)*	*2130 (31.8)*	*4568 (68.2)*
Kinshasa	70 (16.2)	365 (83.9)	87 (21.2)	323 (78.8)	40 (32.3)	84 (67.7)	169 (29.1)	411 (70.9)
Bas Congo	34 (10.9)	281 (89.2)	46 (16.0)	241 (84.0)	40 (33.6)	79 (66.4)	80 (26.0)	228 (74.0)
Bandundu	243 (24.6)	749 (75.5)	262 (27.8)	682 (72.2)	104 (29.8)	245 (70.2)	476 (37.8)	783 (62.2)
Equateur	187 (19.7)	761 (80.2)	199 (21.5)	728 (78.5)	98 (27.3)	261 (72.7)	406 (33.1)	821 (66.9)
Province Orientale	58 (08.1)	657 (91.9)	64 (10.0)	578 (90.0)	51 (18.4)	226 (81.6)	293 (26.5)	811 (73.5)
Maniema	37 (21.9)	132 (78.1)	32 (24.1)	101 (75.9)	13 (18.6)	57 (81.4)	113 (40.8)	164 (59.2)
Kasai Oriental	115 (13.8)	720 (86.2)	139 (18.9)	596 (81.1)	91 (28.1)	233 (71.9)	281 (25.4)	825 (74.6)
Kasai Occidental	151 (32.1)	320 (67.9)	142 (30.8)	319 (69.2)	137 (35.5)	249 (64.5)	312 (37.3)	525 (62.7)

**Table 5:** Logistic Regression Model for ITN utilization.

Variables	Bednets utilization		OR	(95% CI)	*P* value
	No	Yes			
	*N* = 1024	*N* = 3720			
Age					
15–29 years	595 (58.1)	1988 (53.4)	1		
30+	429 (41.9)	1732 (46.6)	0.874	0.746–1.025	>.05
Attended school					
No	278 (27.1)	662 (17.8)	1		
Yes	746 (72.9)	3058 (82.2)	1.322	1.085–1.611	<.05
Urban					
No	385 (37.6)	1327 (35.7)	1		
Yes	639 (62.4)	2393 (64.3)	1.236	1.049–1.458	<.05
Marital status-live in union					
No	159 (15.5)	512 (13.8)	1		
Yes	865 (84.5)	3208 (86.2)	1.019	0.815–1.274	>.05
Knowledge cause of malaria					
No	139 (16.2)	334 (9.5)	1		
Yes	720 (83.8)	3166 (90.5)	1.378	1.09–1.742	<.05
Perception severity					
Low	167 (19.6)	413 (11.9)	1		
High	685 (80.4)	3069 (88.1)	1.189	0.949–1.489	>.05
Perception of the ITN^*∗*^					
Low	257 (30.1)	762 (21.9)	1		
High	597 (69.9)	2725 (78.1)	1.521	1.270–1.821	<.05
Self-efficacy					
Low	154 (18.1)	252 (7.2)	1		
High	698 (81.9)	3242 (92.8)	1.915	1.489–2.464	<.05
Norm social					
No	480 (55.9)	1356 (38.6)	1		
Yes	378 (44.1)	2156 (61.4)	1.930	1.645–2.265	<.05
Attitude: bednets are useful					
No	257 (30.0)	579 (16.5)	1		
Yes	600 (70.0)	2929 (83.5)	1.529	1.258–1.858	<.05

^*∗*^Containing missing values.
